# [1,1′-Bis(diphenyl­phosphan­yl)ferrocene-κ^2^ *P*,*P*′](η^5^-cyclo­penta­dien­yl)(dicyanamido-κ*N*)ruthenium(II) dichloro­methane monosolvate

**DOI:** 10.1107/S1600536812014274

**Published:** 2012-04-13

**Authors:** Libin Gao

**Affiliations:** a Institute of Chemistry and Pharmacy, Qingdao Agricultural University, Qingdao 266109, People’s Republic of China

## Abstract

The title compound, [FeRu(C_5_H_5_)(C_2_N_3_)(C_17_H_14_P)_2_], was obtained by reaction of Cp(dppf)RuCl [dppf = 1,1′-bis­(diphenyl­phosphan­yl)ferrocene] with sodium dicyanamide in dichloro­methane. The Ru^II^ atom is capped by an η^5^-cyclo­penta­dienyl (Cp) ring, a chelating dppf and a terminal C_2_N_3_ unit, giving three-legged piano-stool geometry. The C—N—C angle of the N(CN)_2_ ligand [120.8 (6)°] is significantly smaller than that in the corresponding diruthenium complex [127.2 (9)°; Zhang *et al.* (2003 ▶). *Inorg. Chem.* **42**, 633–640] due to steric hindrance between the two {Cp(PPh_3_)_2_Ru} building blocks. Disorder was found in the dichloro­methane solvent mol­ecule, which was refined as disordered over two positions, with a site-occupancy ratio of 0.53:0.47 (2).

## Related literature

For background to the use of CpRu(dppf), see: Gao *et al.* (2005 ▶). For the structure of [CpRu(dppf)(NCS)], which has a related geometry, see: Lu *et al.* (2004 ▶). For the corresponding diruthenium complex bridged with an N(CN)_2_ ligand, see: Zhang *et al.* (2003 ▶).
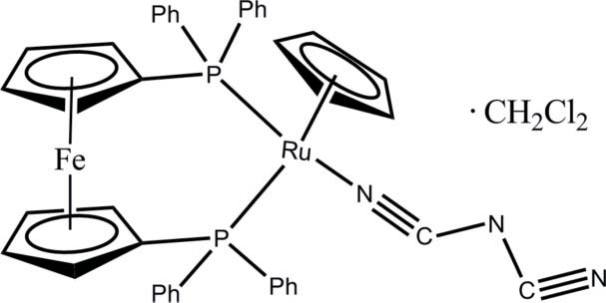


## Experimental

### 

#### Crystal data

[FeRu(C_5_H_5_)(C_2_N_3_)(C_17_H_14_P)_2_]
*M* *_r_* = 871.49
Monoclinic, 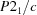

*a* = 10.7858 (2) Å
*b* = 23.2456 (4) Å
*c* = 15.1530 (3) Å
β = 91.916 (1)°
*V* = 3797.07 (12) Å^3^
*Z* = 4
Mo *K*α radiation
μ = 1.04 mm^−1^
*T* = 293 K
1.00 × 0.48 × 0.40 mm

#### Data collection

Bruker SMART CCD diffractometer
Absorption correction: multi-scan (*SADABS*; Sheldrick, 1996 ▶) *T*_min_ = 0.721, *T*_max_ = 1.000
11634 measured reflections
6675 independent reflections
5179 reflections with *I* > 2σ(*I*)
*R*_int_ = 0.029

#### Refinement

*R*[*F* ^2^ > 2σ(*F* ^2^)] = 0.050
*wR*(*F* ^2^) = 0.111
*S* = 1.12
6675 reflections
488 parameters
3 restraints
H-atom parameters constrained
Δρ_max_ = 0.55 e Å^−3^
Δρ_min_ = −0.84 e Å^−3^

### 

Data collection: *SMART* (Bruker, 2007 ▶); cell refinement: *SAINT* (Bruker, 2007 ▶); data reduction: *SAINT*; program(s) used to solve structure: *SHELXTL* (Sheldrick, 2008 ▶); program(s) used to refine structure: *SHELXTL*; molecular graphics: *SHELXTL*; software used to prepare material for publication: *SHELXTL*.

## Supplementary Material

Crystal structure: contains datablock(s) I, global. DOI: 10.1107/S1600536812014274/nk2142sup1.cif

Structure factors: contains datablock(s) I. DOI: 10.1107/S1600536812014274/nk2142Isup2.hkl

Additional supplementary materials:  crystallographic information; 3D view; checkCIF report

## Figures and Tables

**Table 1:** Selected geometric parameters (Å, °)

Ru1—N1	2.071 (4)
Ru1—P2	2.3050 (13)
Ru1—P2	2.3050 (13)
Ru1—P1	2.3164 (13)
Ru1—*Cg*(C21–C25)	1.863 (2)

**Fig. 1.**
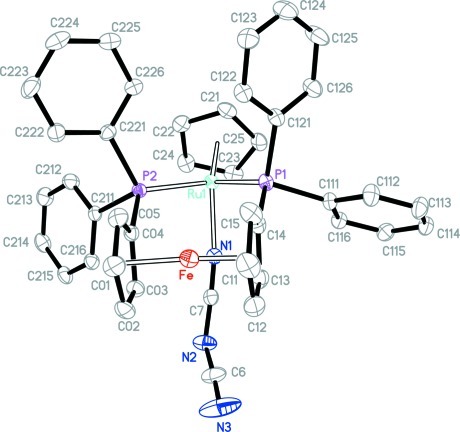
The title compound with 20% displacment ellipsoids. H atoms and solvent CH2Cl2 are not shown clarity.

## supplementary crystallographic information

### Comment

CpRu(dppf) (Gao *et al.*,2005) unit is a good building block for preparing heteronuclear molecular wires with excellent electrochemistry and photophysical properties. The overall geometry of the title compound (Fig. 1) is similar to that of [CpRu(dppf)(NCS)] (Lu *et al.*, 2004), which shows a mononuclear Ru^II^ capped by an η^5^-Cp ring, a chelating η2-dppf and a terminal N(CN)_2_ ligand, thus completing a three-legged piano-stool configuration. The molecule of the title compound is shown in Fig.1 and the selected geometric parameters are given in Table 1. The distances of Ru—N (2.072 (5) Å) is slightly shorter than that found in compound [{Cp(PPh_3_)_2_Ru}_2_ N(CN)_2_](SbF_6_) (Zhang *et al.*, 2003). The angle of the N(CN)_2_ ligand (120.8 (6)°) is significantly smaller than that in compound [{Cp(PPh_3_)_2_Ru}_2_ N(CN)_2_] (SbF_6_) (127.2 (9)°) due to the steric hindrance between the two {Cp(PPh_3_)_2_Ru} building blocks. The P1—Ru1—P2 chelate angle (96.60 (5)°) in the title compound is similar to that in the dppf analogue [CpRu (dppf)(NCS)](96.77 (3)°) (Lu *et al.*, 2004) The overall coordination environment of Ru^II^ ion is a distorted octahedral geometry.

### Experimental

To a dichloromethane (20 ml) solution of Cp(dppf)_2_RuCl (0.20 mmol, 151.6 mg) was added a methanol (5 ml) solution of NaN(CN)_2_ (0.50 mmol, 44.5 mg) with the color change from orange into pale yellow. After the solution was stirred at room temperature for 4 h, the solvents were evaporated *in vacuo* to leave a residue which was dissolved in 3 ml of dichloromethane. After taken by filtration, the filtrate was layered with petroleum ether to give pale yellow crystals of the product. Yield: 83%. Crystals suitable for data collection were obtained by slow evaporation from a dichloromethane and hexane solution at room temperature

### Refinement

H atoms were positioned geometrically, with C—H distances of 0.96 or 0.93 Å, and allowed to ride on their respective parent C atoms, with *U*_iso_(H) = 1.2Ueq(C). Disorder was found in the solvent dichloromethane, which was refined as disordered over two positions, with site occupancy ratio 0.53:0.47 (2)

### Crystal data

**Table:**

[FeRu(C_5_H_5_)(C_2_N_3_)(C_17_H_14_P)_2_]	*F*(000) = 1768
*M**_r_* = 871.49	*D*_x_ = 1.524 Mg m^−^^3^
Monoclinic, *P*2_1_/*c*	Mo *K*α radiation, λ = 0.71073 Å
*a* = 10.7858 (2) Å	Cell parameters from 5840 reflections
*b* = 23.2456 (4) Å	θ = 1.6–25.0°
*c* = 15.1530 (3) Å	µ = 1.04 mm^−^^1^
β = 91.916 (1)°	*T* = 293 K
*V* = 3797.07 (12) Å^3^	Prism, yellow
*Z* = 4	1.00 × 0.48 × 0.40 mm

### Data collection

**Table:**

Bruker SMART CCD diffractometer	6675 independent reflections
Radiation source: fine-focus sealed tube	5179 reflections with *I* > 2σ(*I*)
Graphite monochromator	*R*_int_ = 0.029
phi and ω scans	θ_max_ = 25.0°, θ_min_ = 1.6°
Absorption correction: multi-scan (*SADABS*; Sheldrick, 1996)	*h* = −12→12
*T*_min_ = 0.721, *T*_max_ = 1.000	*k* = −27→23
11634 measured reflections	*l* = −18→13

### Refinement

**Table:**

Refinement on *F*^2^	Primary atom site location: structure-invariant direct methods
Least-squares matrix: full	Secondary atom site location: difference Fourier map
*R*[*F*^2^ > 2σ(*F*^2^)] = 0.050	Hydrogen site location: inferred from neighbouring sites
*wR*(*F*^2^) = 0.111	H-atom parameters constrained
*S* = 1.12	*w* = 1/[σ^2^(*F*_o_^2^) + (0.0223*P*)^2^ + 9.9372*P*] where *P* = (*F*_o_^2^ + 2*F*_c_^2^)/3
6675 reflections	(Δ/σ)_max_ = 0.002
488 parameters	Δρ_max_ = 0.55 e Å^−^^3^
3 restraints	Δρ_min_ = −0.84 e Å^−^^3^

### Special details

**Table:**

Geometry. All e.s.d.'s (except the e.s.d. in the dihedral angle between two l.s. planes) are estimated using the full covariance matrix. The cell e.s.d.'s are taken into account individually in the estimation of e.s.d.'s in distances, angles and torsion angles; correlations between e.s.d.'s in cell parameters are only used when they are defined by crystal symmetry. An approximate (isotropic) treatment of cell e.s.d.'s is used for estimating e.s.d.'s involving l.s. planes.
Refinement. Refinement of *F*^2^ against ALL reflections. The weighted *R*-factor *wR* and goodness of fit *S* are based on *F*^2^, conventional *R*-factors *R* are based on *F*, with *F* set to zero for negative *F*^2^. The threshold expression of *F*^2^ > σ(*F*^2^) is used only for calculating *R*-factors(gt) *etc*. and is not relevant to the choice of reflections for refinement. *R*-factors based on *F*^2^ are statistically about twice as large as those based on *F*, and *R*- factors based on ALL data will be even larger.

### Fractional atomic coordinates and isotropic or equivalent isotropic displacement parameters (Å^2^)

**Table:**

	*x*	*y*	*z*	*U*_iso_*/*U*_eq_	Occ. (<1)
Ru1	0.45845 (4)	0.634892 (17)	0.57549 (2)	0.03596 (12)	
Fe	0.35171 (7)	0.60717 (3)	0.84919 (4)	0.0468 (2)	
P1	0.58633 (12)	0.61849 (6)	0.69878 (8)	0.0396 (3)	
P2	0.29837 (12)	0.67701 (5)	0.64910 (8)	0.0369 (3)	
N1	0.3711 (4)	0.55699 (18)	0.5966 (3)	0.0429 (10)	
N2	0.2399 (5)	0.4731 (2)	0.6204 (4)	0.0723 (15)	
N3	0.2171 (11)	0.4231 (3)	0.7574 (5)	0.167 (4)	
C03	0.1926 (5)	0.5967 (2)	0.7760 (3)	0.0495 (13)	
H03A	0.1755	0.5654	0.7339	0.059*	
C04	0.2479 (4)	0.6511 (2)	0.7555 (3)	0.0404 (11)	
C05	0.2557 (5)	0.6825 (2)	0.8375 (3)	0.0550 (14)	
H05A	0.2906	0.7211	0.8456	0.066*	
C02	0.1668 (5)	0.5957 (3)	0.8671 (4)	0.0602 (15)	
H02A	0.1294	0.5637	0.8987	0.072*	
C01	0.2059 (6)	0.6479 (3)	0.9045 (4)	0.0641 (17)	
H01A	0.2007	0.6586	0.9669	0.077*	
C6	0.2320 (7)	0.4478 (3)	0.6950 (5)	0.078 (2)	
C7	0.3122 (5)	0.5173 (2)	0.6117 (3)	0.0449 (12)	
C11	0.4735 (6)	0.5853 (3)	0.9495 (4)	0.077 (2)	
H11E	0.4625	0.5927	1.0124	0.093*	
C12	0.4347 (6)	0.5359 (3)	0.9036 (4)	0.0730 (19)	
H12C	0.3920	0.5028	0.9287	0.088*	
C13	0.4661 (5)	0.5423 (2)	0.8142 (4)	0.0558 (14)	
H13A	0.4493	0.5143	0.7669	0.067*	
C14	0.5258 (5)	0.5963 (2)	0.8042 (3)	0.0483 (13)	
C15	0.5314 (5)	0.6229 (3)	0.8893 (3)	0.0633 (16)	
H15A	0.5672	0.6606	0.9034	0.076*	
C21	0.5799 (5)	0.6942 (3)	0.5066 (3)	0.0556 (15)	
H21E	0.6363	0.7223	0.5347	0.067*	
C22	0.4560 (6)	0.7048 (3)	0.4777 (3)	0.0553 (14)	
H22B	0.4118	0.7415	0.4826	0.066*	
C23	0.5079 (5)	0.6137 (3)	0.4372 (3)	0.0554 (15)	
H23A	0.5039	0.5754	0.4103	0.066*	
C24	0.4105 (5)	0.6550 (3)	0.4349 (3)	0.0564 (15)	
H24A	0.3288	0.6506	0.4054	0.068*	
C25	0.6121 (5)	0.6378 (3)	0.4802 (3)	0.0551 (14)	
H25A	0.6937	0.6197	0.4882	0.066*	
C111	0.7006 (5)	0.5605 (2)	0.6884 (3)	0.0443 (12)	
C112	0.7948 (5)	0.5537 (3)	0.7530 (4)	0.0618 (16)	
H11D	0.7975	0.5778	0.8019	0.074*	
C113	0.8841 (6)	0.5114 (3)	0.7452 (4)	0.0689 (17)	
H11C	0.9459	0.5071	0.7890	0.083*	
C114	0.8815 (5)	0.4757 (3)	0.6727 (4)	0.0603 (15)	
H11F	0.9428	0.4480	0.6666	0.072*	
C115	0.7879 (5)	0.4812 (2)	0.6093 (4)	0.0569 (15)	
H11B	0.7852	0.4564	0.5611	0.068*	
C116	0.6977 (5)	0.5232 (2)	0.6164 (3)	0.0476 (13)	
H11A	0.6351	0.5266	0.5729	0.057*	
C121	0.6855 (5)	0.6805 (2)	0.7238 (3)	0.0442 (12)	
C122	0.6462 (5)	0.7273 (2)	0.7714 (3)	0.0502 (13)	
H12D	0.5703	0.7258	0.7986	0.060*	
C123	0.7179 (6)	0.7763 (3)	0.7793 (4)	0.0652 (16)	
H12B	0.6909	0.8070	0.8131	0.078*	
C124	0.8274 (7)	0.7802 (3)	0.7380 (4)	0.078 (2)	
H12F	0.8749	0.8134	0.7432	0.094*	
C125	0.8679 (6)	0.7347 (3)	0.6885 (5)	0.085 (2)	
H12E	0.9420	0.7374	0.6591	0.102*	
C126	0.7980 (5)	0.6849 (3)	0.6826 (4)	0.0633 (16)	
H12A	0.8269	0.6539	0.6504	0.076*	
C211	0.1572 (4)	0.6711 (2)	0.5762 (3)	0.0407 (11)	
C212	0.1320 (5)	0.7133 (2)	0.5137 (4)	0.0554 (14)	
H21C	0.1801	0.7465	0.5141	0.066*	
C213	0.0370 (5)	0.7073 (3)	0.4506 (4)	0.0646 (16)	
H21F	0.0211	0.7364	0.4097	0.078*	
C214	−0.0336 (5)	0.6581 (3)	0.4490 (4)	0.0613 (16)	
H21A	−0.0971	0.6537	0.4065	0.074*	
C215	−0.0105 (5)	0.6157 (3)	0.5099 (4)	0.0552 (14)	
H21D	−0.0589	0.5826	0.5092	0.066*	
C216	0.0848 (5)	0.6220 (2)	0.5728 (3)	0.0479 (13)	
H21B	0.1005	0.5926	0.6134	0.057*	
C221	0.3073 (5)	0.7550 (2)	0.6697 (3)	0.0447 (12)	
C222	0.2047 (6)	0.7856 (3)	0.6977 (4)	0.0643 (16)	
H22E	0.1308	0.7661	0.7068	0.077*	
C223	0.2104 (7)	0.8439 (3)	0.7123 (5)	0.079 (2)	
H22F	0.1400	0.8638	0.7288	0.094*	
C224	0.3204 (7)	0.8729 (3)	0.7023 (4)	0.0733 (19)	
H22D	0.3247	0.9123	0.7124	0.088*	
C225	0.4237 (6)	0.8433 (3)	0.6775 (4)	0.0621 (16)	
H22C	0.4986	0.8626	0.6722	0.075*	
C226	0.4169 (5)	0.7846 (2)	0.6603 (3)	0.0485 (13)	
H22A	0.4870	0.7651	0.6423	0.058*	
Cl1	0.950 (3)	0.5700 (14)	1.048 (2)	0.318 (16)	0.47 (2)
Cl2	0.8391 (7)	0.6687 (4)	0.9689 (7)	0.109 (3)	0.47 (2)
C99	0.872 (5)	0.6209 (15)	1.0479 (18)	0.29 (4)	0.47 (2)
H99A	0.9043	0.6438	1.0971	0.351*	0.47 (2)
H99B	0.7919	0.6070	1.0656	0.351*	0.47 (2)
Cl1'	0.8624 (17)	0.5479 (4)	0.9963 (7)	0.175 (5)	0.53 (2)
Cl2'	0.8505 (18)	0.6653 (7)	0.9971 (15)	0.282 (9)	0.53 (2)
C99'	0.821 (2)	0.6008 (10)	1.0370 (18)	0.160 (17)	0.53 (2)
H99C	0.8545	0.6004	1.0973	0.192*	0.53 (2)
H99D	0.7319	0.5979	1.0405	0.192*	0.53 (2)

### Atomic displacement parameters (Å^2^)

**Table:**

	*U*^11^	*U*^22^	*U*^33^	*U*^12^	*U*^13^	*U*^23^
Ru1	0.0352 (2)	0.0436 (2)	0.02893 (19)	−0.00103 (19)	0.00017 (14)	−0.00060 (17)
Fe	0.0538 (5)	0.0545 (5)	0.0325 (4)	−0.0030 (4)	0.0056 (3)	0.0018 (3)
P1	0.0368 (7)	0.0498 (8)	0.0321 (6)	0.0009 (6)	−0.0022 (5)	−0.0040 (5)
P2	0.0369 (7)	0.0363 (7)	0.0375 (7)	−0.0016 (5)	0.0003 (5)	−0.0013 (5)
N1	0.045 (2)	0.045 (2)	0.039 (2)	−0.001 (2)	0.0005 (19)	−0.0042 (19)
N2	0.090 (4)	0.060 (3)	0.068 (3)	−0.030 (3)	0.016 (3)	−0.008 (3)
N3	0.317 (13)	0.086 (5)	0.101 (6)	−0.056 (7)	0.052 (7)	0.024 (5)
C03	0.051 (3)	0.051 (3)	0.047 (3)	−0.003 (3)	0.005 (2)	−0.001 (2)
C04	0.039 (3)	0.042 (3)	0.041 (3)	0.003 (2)	0.005 (2)	−0.003 (2)
C05	0.067 (4)	0.050 (3)	0.048 (3)	0.001 (3)	0.005 (3)	−0.014 (3)
C02	0.058 (4)	0.068 (4)	0.057 (3)	−0.006 (3)	0.022 (3)	0.006 (3)
C01	0.083 (4)	0.065 (4)	0.046 (3)	0.004 (3)	0.027 (3)	−0.002 (3)
C6	0.117 (6)	0.042 (4)	0.078 (5)	−0.014 (4)	0.026 (4)	−0.005 (3)
C7	0.047 (3)	0.046 (3)	0.042 (3)	0.005 (3)	0.005 (2)	−0.009 (2)
C11	0.073 (4)	0.119 (6)	0.040 (3)	0.002 (4)	0.003 (3)	0.017 (4)
C12	0.072 (4)	0.084 (5)	0.063 (4)	0.008 (4)	0.008 (3)	0.031 (4)
C13	0.062 (4)	0.056 (4)	0.049 (3)	0.011 (3)	0.002 (3)	0.013 (3)
C14	0.042 (3)	0.064 (4)	0.038 (3)	0.006 (3)	−0.005 (2)	0.006 (2)
C15	0.059 (4)	0.092 (5)	0.037 (3)	−0.013 (3)	−0.009 (3)	0.001 (3)
C21	0.057 (4)	0.073 (4)	0.038 (3)	−0.016 (3)	0.006 (3)	0.011 (3)
C22	0.069 (4)	0.061 (4)	0.036 (3)	0.001 (3)	0.003 (3)	0.014 (3)
C23	0.071 (4)	0.066 (4)	0.030 (3)	−0.011 (3)	0.010 (3)	−0.009 (2)
C24	0.061 (4)	0.081 (4)	0.027 (3)	0.003 (3)	−0.003 (2)	0.006 (3)
C25	0.049 (3)	0.073 (4)	0.044 (3)	0.004 (3)	0.019 (2)	0.008 (3)
C111	0.039 (3)	0.051 (3)	0.043 (3)	0.006 (2)	−0.002 (2)	−0.003 (2)
C112	0.061 (4)	0.069 (4)	0.054 (3)	0.011 (3)	−0.015 (3)	−0.015 (3)
C113	0.065 (4)	0.067 (4)	0.072 (4)	0.015 (3)	−0.021 (3)	−0.001 (3)
C114	0.053 (4)	0.054 (4)	0.074 (4)	0.013 (3)	0.006 (3)	0.006 (3)
C115	0.066 (4)	0.051 (3)	0.054 (3)	0.005 (3)	0.004 (3)	−0.005 (3)
C116	0.050 (3)	0.049 (3)	0.044 (3)	0.002 (3)	−0.003 (2)	−0.004 (2)
C121	0.042 (3)	0.055 (3)	0.035 (3)	−0.002 (2)	−0.003 (2)	−0.010 (2)
C122	0.050 (3)	0.061 (4)	0.040 (3)	−0.001 (3)	−0.001 (2)	−0.009 (2)
C123	0.077 (4)	0.061 (4)	0.057 (4)	−0.001 (3)	−0.003 (3)	−0.020 (3)
C124	0.078 (5)	0.078 (5)	0.078 (5)	−0.027 (4)	−0.003 (4)	−0.026 (4)
C125	0.058 (4)	0.097 (5)	0.101 (5)	−0.031 (4)	0.017 (4)	−0.031 (5)
C126	0.052 (4)	0.068 (4)	0.070 (4)	−0.006 (3)	0.012 (3)	−0.022 (3)
C211	0.035 (3)	0.043 (3)	0.043 (3)	0.003 (2)	−0.002 (2)	−0.005 (2)
C212	0.049 (3)	0.057 (3)	0.060 (3)	−0.005 (3)	−0.007 (3)	0.011 (3)
C213	0.058 (4)	0.074 (4)	0.061 (4)	0.007 (3)	−0.017 (3)	0.014 (3)
C214	0.044 (3)	0.087 (5)	0.052 (3)	0.001 (3)	−0.011 (3)	0.000 (3)
C215	0.036 (3)	0.065 (4)	0.063 (4)	−0.004 (3)	−0.006 (3)	−0.014 (3)
C216	0.043 (3)	0.050 (3)	0.050 (3)	0.000 (2)	−0.003 (2)	−0.002 (2)
C221	0.050 (3)	0.039 (3)	0.045 (3)	0.000 (2)	−0.006 (2)	−0.003 (2)
C222	0.053 (4)	0.055 (4)	0.085 (4)	−0.001 (3)	0.000 (3)	−0.015 (3)
C223	0.086 (5)	0.051 (4)	0.098 (5)	0.017 (4)	−0.004 (4)	−0.018 (4)
C224	0.114 (6)	0.042 (3)	0.064 (4)	−0.005 (4)	−0.009 (4)	−0.004 (3)
C225	0.077 (4)	0.052 (4)	0.056 (4)	−0.014 (3)	−0.002 (3)	0.001 (3)
C226	0.058 (3)	0.044 (3)	0.043 (3)	−0.002 (3)	−0.001 (2)	0.000 (2)
Cl1	0.28 (2)	0.41 (2)	0.27 (2)	0.215 (19)	0.182 (17)	0.23 (2)
Cl2	0.114 (6)	0.104 (5)	0.106 (5)	−0.040 (4)	−0.026 (3)	0.037 (3)
C99	0.72 (13)	0.067 (17)	0.11 (2)	0.10 (4)	0.19 (5)	0.051 (15)
Cl1'	0.265 (13)	0.124 (6)	0.140 (7)	0.037 (6)	0.077 (7)	0.049 (5)
Cl2'	0.43 (2)	0.211 (13)	0.200 (14)	−0.049 (13)	0.006 (12)	0.005 (10)
C99'	0.14 (2)	0.16 (4)	0.18 (3)	−0.03 (2)	−0.013 (19)	0.11 (3)

### Geometric parameters (Å, º)

**Table:**

Ru1—N1	2.071 (4)	C24—H24A	0.9800
Ru1—C21	2.191 (5)	C25—H25A	0.9794
Ru1—C22	2.200 (5)	C111—C116	1.392 (7)
Ru1—C24	2.225 (5)	C111—C112	1.396 (7)
Ru1—C23	2.234 (5)	C112—C113	1.383 (8)
Ru1—C25	2.234 (5)	C112—H11D	0.9300
Ru1—P2	2.3050 (13)	C113—C114	1.376 (8)
Ru1—P1	2.3164 (13)	C113—H11C	0.9300
Fe—C03	2.027 (5)	C114—C115	1.376 (8)
Fe—C13	2.029 (6)	C114—H11F	0.9300
Fe—C14	2.035 (5)	C115—C116	1.385 (7)
Fe—C05	2.038 (6)	C115—H11B	0.9300
Fe—C01	2.039 (6)	C116—H11A	0.9300
Fe—C02	2.039 (6)	C121—C122	1.379 (7)
Fe—C11	2.040 (6)	C121—C126	1.388 (7)
Fe—C12	2.043 (6)	C122—C123	1.380 (8)
Fe—C15	2.044 (6)	C122—H12D	0.9300
Fe—C04	2.050 (5)	C123—C124	1.358 (9)
P1—C14	1.821 (5)	C123—H12B	0.9300
P1—C121	1.827 (5)	C124—C125	1.377 (9)
P1—C111	1.837 (5)	C124—H12F	0.9300
P2—C04	1.822 (5)	C125—C126	1.382 (8)
P2—C221	1.842 (5)	C125—H12E	0.9300
P2—C211	1.856 (5)	C126—H12A	0.9300
N1—C7	1.148 (6)	C211—C212	1.383 (7)
N2—C6	1.280 (8)	C211—C216	1.384 (7)
N2—C7	1.297 (7)	C212—C213	1.385 (7)
N3—C6	1.123 (9)	C212—H21C	0.9300
C03—C02	1.417 (7)	C213—C214	1.374 (8)
C03—C04	1.436 (7)	C213—H21F	0.9300
C03—H03A	0.9811	C214—C215	1.368 (8)
C04—C05	1.441 (7)	C214—H21A	0.9300
C05—C01	1.414 (8)	C215—C216	1.386 (7)
C05—H05A	0.9791	C215—H21D	0.9300
C02—C01	1.398 (8)	C216—H21B	0.9300
C02—H02A	0.9789	C221—C226	1.380 (7)
C01—H01A	0.9811	C221—C222	1.393 (7)
C11—C12	1.398 (9)	C222—C223	1.375 (8)
C11—C15	1.424 (8)	C222—H22E	0.9300
C11—H11E	0.9796	C223—C224	1.377 (9)
C12—C13	1.414 (8)	C223—H22F	0.9300
C12—H12C	0.9800	C224—C225	1.373 (9)
C13—C14	1.421 (8)	C224—H22D	0.9300
C13—H13A	0.9806	C225—C226	1.390 (7)
C14—C15	1.429 (7)	C225—H22C	0.9300
C15—H15A	0.9784	C226—H22A	0.9300
C21—C22	1.414 (8)	Cl1—C99	1.45 (2)
C21—C25	1.418 (8)	Cl2—C99	1.663 (19)
C21—H21E	0.9802	C99—H99A	0.9700
C22—C24	1.407 (8)	C99—H99B	0.9700
C22—H22B	0.9813	Cl1'—C99'	1.45 (2)
C23—C25	1.397 (8)	Cl2'—C99'	1.651 (18)
C23—C24	1.423 (8)	C99'—H99C	0.9700
C23—H23A	0.9795	C99'—H99D	0.9700
			
N1—Ru1—C21	155.02 (19)	C12—C13—Fe	70.2 (4)
N1—Ru1—C22	139.02 (19)	C14—C13—Fe	69.7 (3)
C21—Ru1—C22	37.6 (2)	C12—C13—H13A	125.9
N1—Ru1—C24	103.84 (19)	C14—C13—H13A	125.5
C21—Ru1—C24	62.3 (2)	Fe—C13—H13A	125.8
C22—Ru1—C24	37.1 (2)	C13—C14—C15	106.9 (5)
N1—Ru1—C23	94.37 (18)	C13—C14—P1	121.4 (4)
C21—Ru1—C23	61.7 (2)	C15—C14—P1	131.6 (4)
C22—Ru1—C23	61.9 (2)	C13—C14—Fe	69.3 (3)
C24—Ru1—C23	37.2 (2)	C15—C14—Fe	69.9 (3)
N1—Ru1—C25	118.49 (19)	P1—C14—Fe	128.4 (3)
C21—Ru1—C25	37.4 (2)	C11—C15—C14	107.8 (6)
C22—Ru1—C25	62.3 (2)	C11—C15—Fe	69.4 (4)
C24—Ru1—C25	61.9 (2)	C14—C15—Fe	69.1 (3)
C23—Ru1—C25	36.4 (2)	C11—C15—H15A	126.1
N1—Ru1—P2	86.83 (12)	C14—C15—H15A	126.1
C21—Ru1—P2	115.62 (17)	Fe—C15—H15A	126.0
C22—Ru1—P2	91.14 (16)	C22—C21—C25	108.1 (5)
C24—Ru1—P2	102.70 (16)	C22—C21—Ru1	71.6 (3)
C23—Ru1—P2	138.90 (16)	C25—C21—Ru1	73.0 (3)
C25—Ru1—P2	152.06 (16)	C22—C21—H21E	125.8
N1—Ru1—P1	89.63 (11)	C25—C21—H21E	125.8
C21—Ru1—P1	97.96 (15)	Ru1—C21—H21E	125.8
C22—Ru1—P1	131.18 (16)	C24—C22—C21	108.1 (5)
C24—Ru1—P1	156.91 (16)	C24—C22—Ru1	72.4 (3)
C23—Ru1—P1	124.46 (16)	C21—C22—Ru1	70.9 (3)
C25—Ru1—P1	95.25 (15)	C24—C22—H22B	125.9
P2—Ru1—P1	96.61 (5)	C21—C22—H22B	125.8
C03—Fe—C13	106.2 (2)	Ru1—C22—H22B	125.9
C03—Fe—C14	125.2 (2)	C25—C23—C24	108.8 (5)
C13—Fe—C14	40.9 (2)	C25—C23—Ru1	71.8 (3)
C03—Fe—C05	68.7 (2)	C24—C23—Ru1	71.0 (3)
C13—Fe—C05	157.9 (2)	C25—C23—H23A	125.6
C14—Fe—C05	123.3 (2)	C24—C23—H23A	125.5
C03—Fe—C01	68.3 (2)	Ru1—C23—H23A	125.6
C13—Fe—C01	159.3 (2)	C22—C24—C23	107.3 (5)
C14—Fe—C01	158.2 (2)	C22—C24—Ru1	70.5 (3)
C05—Fe—C01	40.6 (2)	C23—C24—Ru1	71.7 (3)
C03—Fe—C02	40.8 (2)	C22—C24—H24A	126.2
C13—Fe—C02	122.9 (2)	C23—C24—H24A	126.3
C14—Fe—C02	161.1 (2)	Ru1—C24—H24A	126.3
C05—Fe—C02	68.1 (2)	C23—C25—C21	107.5 (5)
C01—Fe—C02	40.1 (2)	C23—C25—Ru1	71.8 (3)
C03—Fe—C11	152.9 (3)	C21—C25—Ru1	69.6 (3)
C13—Fe—C11	68.1 (3)	C23—C25—H25A	126.2
C14—Fe—C11	68.9 (2)	C21—C25—H25A	126.2
C05—Fe—C11	126.3 (3)	Ru1—C25—H25A	126.1
C01—Fe—C11	107.2 (3)	C116—C111—C112	118.4 (5)
C02—Fe—C11	118.5 (2)	C116—C111—P1	121.8 (4)
C03—Fe—C12	118.4 (3)	C112—C111—P1	119.8 (4)
C13—Fe—C12	40.6 (2)	C113—C112—C111	120.9 (5)
C14—Fe—C12	68.8 (2)	C113—C112—H11D	119.6
C05—Fe—C12	161.0 (2)	C111—C112—H11D	119.6
C01—Fe—C12	122.9 (2)	C114—C113—C112	120.0 (5)
C02—Fe—C12	104.9 (3)	C114—C113—H11C	120.0
C11—Fe—C12	40.0 (3)	C112—C113—H11C	120.0
C03—Fe—C15	163.9 (2)	C113—C114—C115	119.8 (5)
C13—Fe—C15	68.4 (3)	C113—C114—H11F	120.1
C14—Fe—C15	41.0 (2)	C115—C114—H11F	120.1
C05—Fe—C15	110.2 (3)	C114—C115—C116	120.8 (5)
C01—Fe—C15	122.1 (3)	C114—C115—H11B	119.6
C02—Fe—C15	154.9 (2)	C116—C115—H11B	119.6
C11—Fe—C15	40.8 (2)	C115—C116—C111	120.1 (5)
C12—Fe—C15	68.1 (3)	C115—C116—H11A	119.9
C03—Fe—C04	41.25 (19)	C111—C116—H11A	119.9
C13—Fe—C04	120.8 (2)	C122—C121—C126	117.8 (5)
C14—Fe—C04	108.57 (19)	C122—C121—P1	122.9 (4)
C05—Fe—C04	41.27 (19)	C126—C121—P1	118.6 (4)
C01—Fe—C04	69.1 (2)	C121—C122—C123	121.1 (5)
C02—Fe—C04	69.1 (2)	C121—C122—H12D	119.5
C11—Fe—C04	164.4 (3)	C123—C122—H12D	119.5
C12—Fe—C04	154.8 (3)	C124—C123—C122	120.5 (6)
C15—Fe—C04	127.4 (2)	C124—C123—H12B	119.8
C14—P1—C121	105.4 (2)	C122—C123—H12B	119.8
C14—P1—C111	97.4 (2)	C123—C124—C125	119.8 (6)
C121—P1—C111	102.0 (2)	C123—C124—H12F	120.1
C14—P1—Ru1	122.21 (17)	C125—C124—H12F	120.1
C121—P1—Ru1	111.49 (17)	C124—C125—C126	119.8 (6)
C111—P1—Ru1	115.75 (16)	C124—C125—H12E	120.1
C04—P2—C221	101.0 (2)	C126—C125—H12E	120.1
C04—P2—C211	103.9 (2)	C125—C126—C121	121.0 (6)
C221—P2—C211	102.1 (2)	C125—C126—H12A	119.5
C04—P2—Ru1	122.59 (16)	C121—C126—H12A	119.5
C221—P2—Ru1	117.67 (18)	C212—C211—C216	117.5 (5)
C211—P2—Ru1	107.06 (16)	C212—C211—P2	119.6 (4)
C7—N1—Ru1	172.6 (4)	C216—C211—P2	122.2 (4)
C6—N2—C7	120.8 (6)	C211—C212—C213	121.7 (5)
C02—C03—C04	108.8 (5)	C211—C212—H21C	119.2
C02—C03—Fe	70.1 (3)	C213—C212—H21C	119.2
C04—C03—Fe	70.3 (3)	C214—C213—C212	119.5 (5)
C02—C03—H03A	125.6	C214—C213—H21F	120.2
C04—C03—H03A	125.7	C212—C213—H21F	120.2
Fe—C03—H03A	125.6	C215—C214—C213	119.9 (5)
C03—C04—C05	105.8 (4)	C215—C214—H21A	120.0
C03—C04—P2	128.4 (4)	C213—C214—H21A	120.0
C05—C04—P2	125.8 (4)	C214—C215—C216	120.2 (5)
C03—C04—Fe	68.5 (3)	C214—C215—H21D	119.9
C05—C04—Fe	68.9 (3)	C216—C215—H21D	119.9
P2—C04—Fe	127.3 (3)	C211—C216—C215	121.1 (5)
C01—C05—C04	108.5 (5)	C211—C216—H21B	119.4
C01—C05—Fe	69.7 (3)	C215—C216—H21B	119.4
C04—C05—Fe	69.8 (3)	C226—C221—C222	118.0 (5)
C01—C05—H05A	125.8	C226—C221—P2	120.9 (4)
C04—C05—H05A	125.7	C222—C221—P2	121.1 (4)
Fe—C05—H05A	125.7	C223—C222—C221	121.3 (6)
C01—C02—C03	108.3 (5)	C223—C222—H22E	119.3
C01—C02—Fe	69.9 (3)	C221—C222—H22E	119.3
C03—C02—Fe	69.1 (3)	C222—C223—C224	120.0 (6)
C01—C02—H02A	125.8	C222—C223—H22F	120.0
C03—C02—H02A	125.9	C224—C223—H22F	120.0
Fe—C02—H02A	125.8	C225—C224—C223	119.6 (6)
C02—C01—C05	108.6 (5)	C225—C224—H22D	120.2
C02—C01—Fe	70.0 (3)	C223—C224—H22D	120.2
C05—C01—Fe	69.7 (3)	C224—C225—C226	120.4 (6)
C02—C01—H01A	125.8	C224—C225—H22C	119.8
C05—C01—H01A	125.6	C226—C225—H22C	119.8
Fe—C01—H01A	125.7	C221—C226—C225	120.7 (5)
N3—C6—N2	174.3 (10)	C221—C226—H22A	119.7
N1—C7—N2	173.8 (6)	C225—C226—H22A	119.7
C12—C11—C15	108.4 (6)	Cl1—C99—Cl2	131.1 (15)
C12—C11—Fe	70.1 (4)	Cl1—C99—H99A	104.5
C15—C11—Fe	69.8 (3)	Cl2—C99—H99A	104.5
C12—C11—H11E	125.8	Cl1—C99—H99B	104.5
C15—C11—H11E	125.9	Cl2—C99—H99B	104.5
Fe—C11—H11E	125.8	H99A—C99—H99B	105.6
C11—C12—C13	108.2 (6)	Cl1'—C99'—Cl2'	123.3 (17)
C11—C12—Fe	69.9 (4)	Cl1'—C99'—H99C	106.5
C13—C12—Fe	69.2 (3)	Cl2'—C99'—H99C	106.5
C11—C12—H12C	126.0	Cl1'—C99'—H99D	106.5
C13—C12—H12C	125.8	Cl2'—C99'—H99D	106.5
Fe—C12—H12C	126.0	H99C—C99'—H99D	106.5
C12—C13—C14	108.6 (6)		
			
N1—Ru1—P1—C14	−48.8 (2)	C05—Fe—C13—C12	171.9 (6)
C21—Ru1—P1—C14	155.1 (3)	C01—Fe—C13—C12	−43.7 (9)
C22—Ru1—P1—C14	135.4 (3)	C02—Fe—C13—C12	−73.6 (5)
C24—Ru1—P1—C14	−175.2 (5)	C11—Fe—C13—C12	37.0 (4)
C23—Ru1—P1—C14	−143.8 (3)	C15—Fe—C13—C12	81.1 (4)
C25—Ru1—P1—C14	−167.3 (3)	C04—Fe—C13—C12	−157.3 (4)
P2—Ru1—P1—C14	38.0 (2)	C03—Fe—C13—C14	125.5 (3)
N1—Ru1—P1—C121	−174.6 (2)	C05—Fe—C13—C14	52.2 (7)
C21—Ru1—P1—C121	29.3 (2)	C01—Fe—C13—C14	−163.3 (6)
C22—Ru1—P1—C121	9.5 (3)	C02—Fe—C13—C14	166.8 (3)
C24—Ru1—P1—C121	58.9 (5)	C11—Fe—C13—C14	−82.6 (4)
C23—Ru1—P1—C121	90.4 (3)	C12—Fe—C13—C14	−119.6 (5)
C25—Ru1—P1—C121	66.8 (2)	C15—Fe—C13—C14	−38.5 (3)
P2—Ru1—P1—C121	−87.82 (18)	C04—Fe—C13—C14	83.0 (3)
N1—Ru1—P1—C111	69.5 (2)	C12—C13—C14—C15	0.4 (6)
C21—Ru1—P1—C111	−86.6 (3)	Fe—C13—C14—C15	60.0 (4)
C22—Ru1—P1—C111	−106.4 (3)	C12—C13—C14—P1	177.2 (4)
C24—Ru1—P1—C111	−57.0 (5)	Fe—C13—C14—P1	−123.2 (4)
C23—Ru1—P1—C111	−25.6 (3)	C12—C13—C14—Fe	−59.6 (4)
C25—Ru1—P1—C111	−49.1 (2)	C121—P1—C14—C13	−166.6 (4)
P2—Ru1—P1—C111	156.26 (19)	C111—P1—C14—C13	−61.9 (5)
N1—Ru1—P2—C04	49.5 (2)	Ru1—P1—C14—C13	64.9 (5)
C21—Ru1—P2—C04	−141.9 (2)	C121—P1—C14—C15	9.3 (6)
C22—Ru1—P2—C04	−171.5 (2)	C111—P1—C14—C15	114.0 (5)
C24—Ru1—P2—C04	152.9 (2)	Ru1—P1—C14—C15	−119.2 (5)
C23—Ru1—P2—C04	142.5 (3)	C121—P1—C14—Fe	106.0 (4)
C25—Ru1—P2—C04	−154.3 (4)	C111—P1—C14—Fe	−149.4 (4)
P1—Ru1—P2—C04	−39.8 (2)	Ru1—P1—C14—Fe	−22.6 (5)
N1—Ru1—P2—C221	175.7 (2)	C03—Fe—C14—C13	−73.1 (4)
C21—Ru1—P2—C221	−15.6 (2)	C05—Fe—C14—C13	−159.2 (3)
C22—Ru1—P2—C221	−45.2 (2)	C01—Fe—C14—C13	164.2 (6)
C24—Ru1—P2—C221	−80.8 (2)	C02—Fe—C14—C13	−36.3 (8)
C23—Ru1—P2—C221	−91.2 (3)	C11—Fe—C14—C13	80.5 (4)
C25—Ru1—P2—C221	−28.0 (4)	C12—Fe—C14—C13	37.4 (4)
P1—Ru1—P2—C221	86.49 (18)	C15—Fe—C14—C13	118.0 (5)
N1—Ru1—P2—C211	−70.1 (2)	C04—Fe—C14—C13	−115.9 (3)
C21—Ru1—P2—C211	98.5 (2)	C03—Fe—C14—C15	168.9 (3)
C22—Ru1—P2—C211	68.9 (2)	C13—Fe—C14—C15	−118.0 (5)
C24—Ru1—P2—C211	33.4 (2)	C05—Fe—C14—C15	82.8 (4)
C23—Ru1—P2—C211	22.9 (3)	C01—Fe—C14—C15	46.2 (7)
C25—Ru1—P2—C211	86.1 (4)	C02—Fe—C14—C15	−154.3 (7)
P1—Ru1—P2—C211	−159.37 (17)	C11—Fe—C14—C15	−37.5 (4)
C21—Ru1—N1—C7	−148 (3)	C12—Fe—C14—C15	−80.6 (4)
C22—Ru1—N1—C7	−81 (3)	C04—Fe—C14—C15	126.1 (3)
C24—Ru1—N1—C7	−95 (3)	C03—Fe—C14—P1	41.2 (5)
C23—Ru1—N1—C7	−131 (3)	C13—Fe—C14—P1	114.3 (5)
C25—Ru1—N1—C7	−160 (3)	C05—Fe—C14—P1	−44.9 (5)
P2—Ru1—N1—C7	8 (3)	C01—Fe—C14—P1	−81.5 (7)
P1—Ru1—N1—C7	104 (3)	C02—Fe—C14—P1	78.0 (8)
C13—Fe—C03—C02	121.9 (4)	C11—Fe—C14—P1	−165.2 (5)
C14—Fe—C03—C02	162.7 (3)	C12—Fe—C14—P1	151.7 (5)
C05—Fe—C03—C02	−80.8 (4)	C15—Fe—C14—P1	−127.7 (6)
C01—Fe—C03—C02	−37.0 (3)	C04—Fe—C14—P1	−1.6 (4)
C11—Fe—C03—C02	48.5 (7)	C12—C11—C15—C14	1.0 (7)
C12—Fe—C03—C02	79.8 (4)	Fe—C11—C15—C14	−58.6 (4)
C15—Fe—C03—C02	−170.2 (8)	C12—C11—C15—Fe	59.7 (5)
C04—Fe—C03—C02	−119.6 (5)	C13—C14—C15—C11	−0.9 (6)
C13—Fe—C03—C04	−118.5 (3)	P1—C14—C15—C11	−177.2 (4)
C14—Fe—C03—C04	−77.7 (4)	Fe—C14—C15—C11	58.8 (4)
C05—Fe—C03—C04	38.8 (3)	C13—C14—C15—Fe	−59.7 (4)
C01—Fe—C03—C04	82.6 (3)	P1—C14—C15—Fe	124.0 (5)
C02—Fe—C03—C04	119.6 (5)	C03—Fe—C15—C11	−154.2 (8)
C11—Fe—C03—C04	168.0 (5)	C13—Fe—C15—C11	−81.1 (4)
C12—Fe—C03—C04	−160.7 (3)	C14—Fe—C15—C11	−119.5 (6)
C15—Fe—C03—C04	−50.6 (10)	C05—Fe—C15—C11	122.5 (4)
C02—C03—C04—C05	0.6 (6)	C01—Fe—C15—C11	78.9 (5)
Fe—C03—C04—C05	−59.1 (4)	C02—Fe—C15—C11	41.1 (8)
C02—C03—C04—P2	−179.1 (4)	C12—Fe—C15—C11	−37.2 (4)
Fe—C03—C04—P2	121.2 (4)	C04—Fe—C15—C11	166.0 (4)
C02—C03—C04—Fe	59.7 (4)	C03—Fe—C15—C14	−34.7 (11)
C221—P2—C04—C03	161.8 (5)	C13—Fe—C15—C14	38.5 (3)
C211—P2—C04—C03	56.2 (5)	C05—Fe—C15—C14	−117.9 (3)
Ru1—P2—C04—C03	−64.9 (5)	C01—Fe—C15—C14	−161.5 (3)
C221—P2—C04—C05	−17.8 (5)	C02—Fe—C15—C14	160.6 (5)
C211—P2—C04—C05	−123.4 (5)	C11—Fe—C15—C14	119.5 (6)
Ru1—P2—C04—C05	115.5 (4)	C12—Fe—C15—C14	82.4 (4)
C221—P2—C04—Fe	−107.4 (3)	C04—Fe—C15—C14	−74.5 (4)
C211—P2—C04—Fe	147.1 (3)	N1—Ru1—C21—C22	98.1 (5)
Ru1—P2—C04—Fe	26.0 (4)	C24—Ru1—C21—C22	37.3 (3)
C13—Fe—C04—C03	79.2 (3)	C23—Ru1—C21—C22	79.7 (4)
C14—Fe—C04—C03	122.6 (3)	C25—Ru1—C21—C22	116.4 (5)
C05—Fe—C04—C03	−117.7 (4)	P2—Ru1—C21—C22	−54.0 (4)
C01—Fe—C04—C03	−80.5 (3)	P1—Ru1—C21—C22	−155.3 (3)
C02—Fe—C04—C03	−37.5 (3)	N1—Ru1—C21—C25	−18.3 (6)
C11—Fe—C04—C03	−159.5 (8)	C22—Ru1—C21—C25	−116.4 (5)
C12—Fe—C04—C03	43.1 (6)	C24—Ru1—C21—C25	−79.1 (4)
C15—Fe—C04—C03	164.4 (3)	C23—Ru1—C21—C25	−36.8 (3)
C03—Fe—C04—C05	117.7 (4)	P2—Ru1—C21—C25	−170.5 (3)
C13—Fe—C04—C05	−163.0 (3)	P1—Ru1—C21—C25	88.2 (3)
C14—Fe—C04—C05	−119.7 (3)	C25—C21—C22—C24	1.0 (6)
C01—Fe—C04—C05	37.2 (3)	Ru1—C21—C22—C24	−63.3 (4)
C02—Fe—C04—C05	80.3 (3)	C25—C21—C22—Ru1	64.3 (3)
C11—Fe—C04—C05	−41.7 (9)	N1—Ru1—C22—C24	−23.3 (5)
C12—Fe—C04—C05	160.9 (5)	C21—Ru1—C22—C24	117.1 (5)
C15—Fe—C04—C05	−77.9 (4)	C23—Ru1—C22—C24	37.8 (3)
C03—Fe—C04—P2	−122.6 (4)	C25—Ru1—C22—C24	79.2 (4)
C13—Fe—C04—P2	−43.4 (4)	P2—Ru1—C22—C24	−109.8 (3)
C14—Fe—C04—P2	0.0 (4)	P1—Ru1—C22—C24	150.4 (3)
C05—Fe—C04—P2	119.7 (5)	N1—Ru1—C22—C21	−140.4 (3)
C01—Fe—C04—P2	156.9 (4)	C24—Ru1—C22—C21	−117.1 (5)
C02—Fe—C04—P2	−160.1 (4)	C23—Ru1—C22—C21	−79.3 (4)
C11—Fe—C04—P2	77.9 (9)	C25—Ru1—C22—C21	−37.9 (3)
C12—Fe—C04—P2	−79.4 (6)	P2—Ru1—C22—C21	133.1 (3)
C15—Fe—C04—P2	41.8 (4)	P1—Ru1—C22—C21	33.3 (4)
C03—C04—C05—C01	−0.3 (6)	N1—Ru1—C23—C25	−134.6 (4)
P2—C04—C05—C01	179.4 (4)	C21—Ru1—C23—C25	37.7 (3)
Fe—C04—C05—C01	−59.1 (4)	C22—Ru1—C23—C25	80.6 (4)
C03—C04—C05—Fe	58.9 (3)	C24—Ru1—C23—C25	118.2 (5)
P2—C04—C05—Fe	−121.5 (4)	P2—Ru1—C23—C25	135.2 (3)
C03—Fe—C05—C01	81.0 (4)	P1—Ru1—C23—C25	−42.0 (4)
C13—Fe—C05—C01	161.6 (6)	N1—Ru1—C23—C24	107.2 (3)
C14—Fe—C05—C01	−160.1 (3)	C21—Ru1—C23—C24	−80.5 (4)
C02—Fe—C05—C01	37.0 (4)	C22—Ru1—C23—C24	−37.6 (3)
C11—Fe—C05—C01	−73.0 (5)	C25—Ru1—C23—C24	−118.2 (5)
C12—Fe—C05—C01	−34.8 (10)	P2—Ru1—C23—C24	17.0 (4)
C15—Fe—C05—C01	−116.1 (4)	P1—Ru1—C23—C24	−160.2 (3)
C04—Fe—C05—C01	119.8 (5)	C21—C22—C24—C23	−0.4 (6)
C03—Fe—C05—C04	−38.8 (3)	Ru1—C22—C24—C23	−62.7 (4)
C13—Fe—C05—C04	41.8 (8)	C21—C22—C24—Ru1	62.3 (3)
C14—Fe—C05—C04	80.1 (4)	C25—C23—C24—C22	−0.3 (6)
C01—Fe—C05—C04	−119.8 (5)	Ru1—C23—C24—C22	61.9 (3)
C02—Fe—C05—C04	−82.8 (3)	C25—C23—C24—Ru1	−62.2 (4)
C11—Fe—C05—C04	167.2 (3)	N1—Ru1—C24—C22	164.5 (3)
C12—Fe—C05—C04	−154.6 (8)	C21—Ru1—C24—C22	−37.8 (3)
C15—Fe—C05—C04	124.1 (3)	C23—Ru1—C24—C22	−116.7 (5)
C04—C03—C02—C01	−0.7 (7)	C25—Ru1—C24—C22	−80.3 (4)
Fe—C03—C02—C01	59.1 (4)	P2—Ru1—C24—C22	74.7 (3)
C04—C03—C02—Fe	−59.8 (4)	P1—Ru1—C24—C22	−71.4 (6)
C03—Fe—C02—C01	−119.8 (5)	N1—Ru1—C24—C23	−78.8 (3)
C13—Fe—C02—C01	164.2 (3)	C21—Ru1—C24—C23	78.9 (4)
C14—Fe—C02—C01	−168.4 (6)	C22—Ru1—C24—C23	116.7 (5)
C05—Fe—C02—C01	−37.5 (3)	C25—Ru1—C24—C23	36.4 (3)
C11—Fe—C02—C01	83.0 (4)	P2—Ru1—C24—C23	−168.6 (3)
C12—Fe—C02—C01	123.9 (4)	P1—Ru1—C24—C23	45.3 (6)
C15—Fe—C02—C01	53.8 (7)	C24—C23—C25—C21	1.0 (6)
C04—Fe—C02—C01	−81.9 (4)	Ru1—C23—C25—C21	−60.8 (3)
C13—Fe—C02—C03	−76.1 (4)	C24—C23—C25—Ru1	61.7 (4)
C14—Fe—C02—C03	−48.6 (8)	C22—C21—C25—C23	−1.2 (6)
C05—Fe—C02—C03	82.3 (3)	Ru1—C21—C25—C23	62.1 (3)
C01—Fe—C02—C03	119.8 (5)	C22—C21—C25—Ru1	−63.4 (3)
C11—Fe—C02—C03	−157.2 (4)	N1—Ru1—C25—C23	53.9 (4)
C12—Fe—C02—C03	−116.3 (4)	C21—Ru1—C25—C23	−117.4 (5)
C15—Fe—C02—C03	173.6 (5)	C22—Ru1—C25—C23	−79.4 (4)
C04—Fe—C02—C03	37.9 (3)	C24—Ru1—C25—C23	−37.2 (3)
C03—C02—C01—C05	0.5 (7)	P2—Ru1—C25—C23	−98.8 (4)
Fe—C02—C01—C05	59.1 (4)	P1—Ru1—C25—C23	146.3 (3)
C03—C02—C01—Fe	−58.6 (4)	N1—Ru1—C25—C21	171.3 (3)
C04—C05—C01—C02	−0.1 (7)	C22—Ru1—C25—C21	38.1 (3)
Fe—C05—C01—C02	−59.3 (4)	C24—Ru1—C25—C21	80.2 (4)
C04—C05—C01—Fe	59.2 (4)	C23—Ru1—C25—C21	117.4 (5)
C03—Fe—C01—C02	37.6 (3)	P2—Ru1—C25—C21	18.6 (5)
C13—Fe—C01—C02	−40.5 (8)	P1—Ru1—C25—C21	−96.2 (3)
C14—Fe—C01—C02	169.9 (5)	C14—P1—C111—C116	121.8 (5)
C05—Fe—C01—C02	119.8 (5)	C121—P1—C111—C116	−130.7 (4)
C11—Fe—C01—C02	−114.0 (4)	Ru1—P1—C111—C116	−9.5 (5)
C12—Fe—C01—C02	−73.0 (5)	C14—P1—C111—C112	−59.4 (5)
C15—Fe—C01—C02	−156.2 (3)	C121—P1—C111—C112	48.2 (5)
C04—Fe—C01—C02	82.0 (4)	Ru1—P1—C111—C112	169.4 (4)
C03—Fe—C01—C05	−82.2 (4)	C116—C111—C112—C113	0.9 (9)
C13—Fe—C01—C05	−160.4 (6)	P1—C111—C112—C113	−178.0 (5)
C14—Fe—C01—C05	50.0 (7)	C111—C112—C113—C114	0.5 (10)
C02—Fe—C01—C05	−119.8 (5)	C112—C113—C114—C115	−1.8 (10)
C11—Fe—C01—C05	126.2 (4)	C113—C114—C115—C116	1.6 (9)
C12—Fe—C01—C05	167.2 (4)	C114—C115—C116—C111	−0.2 (8)
C15—Fe—C01—C05	84.0 (4)	C112—C111—C116—C115	−1.0 (8)
C04—Fe—C01—C05	−37.8 (3)	P1—C111—C116—C115	177.8 (4)
C7—N2—C6—N3	171 (9)	C14—P1—C121—C122	−51.4 (5)
Ru1—N1—C7—N2	86 (6)	C111—P1—C121—C122	−152.7 (4)
C6—N2—C7—N1	−170 (5)	Ru1—P1—C121—C122	83.2 (4)
C03—Fe—C11—C12	45.3 (7)	C14—P1—C121—C126	138.5 (5)
C13—Fe—C11—C12	−37.5 (4)	C111—P1—C121—C126	37.3 (5)
C14—Fe—C11—C12	−81.7 (4)	Ru1—P1—C121—C126	−86.8 (5)
C05—Fe—C11—C12	161.8 (4)	C126—C121—C122—C123	−1.4 (8)
C01—Fe—C11—C12	121.1 (4)	P1—C121—C122—C123	−171.6 (4)
C02—Fe—C11—C12	79.1 (4)	C121—C122—C123—C124	1.9 (9)
C15—Fe—C11—C12	−119.4 (6)	C122—C123—C124—C125	−0.5 (11)
C04—Fe—C11—C12	−165.2 (7)	C123—C124—C125—C126	−1.3 (11)
C03—Fe—C11—C15	164.7 (5)	C124—C125—C126—C121	1.8 (11)
C13—Fe—C11—C15	81.9 (4)	C122—C121—C126—C125	−0.4 (9)
C14—Fe—C11—C15	37.7 (4)	P1—C121—C126—C125	170.2 (5)
C05—Fe—C11—C15	−78.8 (5)	C04—P2—C211—C212	141.9 (4)
C01—Fe—C11—C15	−119.5 (4)	C221—P2—C211—C212	37.1 (5)
C02—Fe—C11—C15	−161.5 (4)	Ru1—P2—C211—C212	−87.1 (4)
C12—Fe—C11—C15	119.4 (6)	C04—P2—C211—C216	−47.7 (5)
C04—Fe—C11—C15	−45.8 (10)	C221—P2—C211—C216	−152.5 (4)
C15—C11—C12—C13	−0.8 (8)	Ru1—P2—C211—C216	83.3 (4)
Fe—C11—C12—C13	58.7 (4)	C216—C211—C212—C213	0.9 (8)
C15—C11—C12—Fe	−59.5 (5)	P2—C211—C212—C213	171.7 (5)
C03—Fe—C12—C11	−158.4 (4)	C211—C212—C213—C214	−0.7 (9)
C13—Fe—C12—C11	119.8 (6)	C212—C213—C214—C215	0.6 (9)
C14—Fe—C12—C11	82.1 (4)	C213—C214—C215—C216	−0.7 (9)
C05—Fe—C12—C11	−50.8 (10)	C212—C211—C216—C215	−1.0 (8)
C01—Fe—C12—C11	−77.1 (5)	P2—C211—C216—C215	−171.6 (4)
C02—Fe—C12—C11	−116.7 (4)	C214—C215—C216—C211	0.9 (8)
C15—Fe—C12—C11	37.8 (4)	C04—P2—C221—C226	121.5 (4)
C04—Fe—C12—C11	170.8 (5)	C211—P2—C221—C226	−131.6 (4)
C03—Fe—C12—C13	81.8 (4)	Ru1—P2—C221—C226	−14.7 (5)
C14—Fe—C12—C13	−37.7 (4)	C04—P2—C221—C222	−56.5 (5)
C05—Fe—C12—C13	−170.6 (7)	C211—P2—C221—C222	50.4 (5)
C01—Fe—C12—C13	163.1 (4)	Ru1—P2—C221—C222	167.3 (4)
C02—Fe—C12—C13	123.5 (4)	C226—C221—C222—C223	2.6 (9)
C11—Fe—C12—C13	−119.8 (6)	P2—C221—C222—C223	−179.3 (5)
C15—Fe—C12—C13	−81.9 (4)	C221—C222—C223—C224	−2.5 (10)
C04—Fe—C12—C13	51.0 (7)	C222—C223—C224—C225	0.3 (10)
C11—C12—C13—C14	0.3 (7)	C223—C224—C225—C226	1.6 (9)
Fe—C12—C13—C14	59.4 (4)	C222—C221—C226—C225	−0.7 (8)
C11—C12—C13—Fe	−59.1 (5)	P2—C221—C226—C225	−178.8 (4)
C03—Fe—C13—C12	−114.9 (4)	C224—C225—C226—C221	−1.4 (8)
C14—Fe—C13—C12	119.6 (5)		
